# Progress in Diseases of the Heart

**Published:** 1904-02-13

**Authors:** 


### PROGRESS IN DISEASES OF THE HEART.

IN an exceedingly practical lecture Pye-Smith[1] laid down the points which should guide us in the prognosis of heart disease. Diastolic and presystolic murmurs are always due to organic disease, as is also an apical systolic murmur heard well round in the axilla. Again, it is very rare for an organic murmur to be idiopathic or primary without an ascertained origin or cause. The least serious of valvular lesions is aortic obstruction; next comes mitral obstruction; then mitral regurgitation; and most serious of all aortic regurgitation. The prognosis of valvular disease when more than one orifice is affected follows the graver lesion of the two. Most cases of a contracted mitral valve end with more or less regurgitation; all aortic cases, if not cut short, end first in mitral and then in tricuspid regurgitation. Contraction of the tricuspid valve is very seldom found without contraction of the mitral also. If the kidneys are diseased, we are debarred from using mercury and digitalis; but if the specific gravity of the urine be high albuminuria generally means only congestion, and these drugs and opium may be used. While bronchitis is very harmful to cardiac cases, phthisis has scarcely any effect. Rheumatism is the most favourable origin of valvular disease; atheroma much less so; a congenital origin takes a third place; septic endocarditis is the most fatal of all. Amongst the most dangerous cases are those with all the symptoms of mitral regurgitation—dropsy and weak irregular pulse—but no murmur. They are mostly in children and depend on an adherent pericardium. The best element in prognosis is the absence of symptoms, and especially is this so with no physical signs of compensatory hypertrophy. The worst is marked evidence of compensatory hypertrophy with symptoms of failure. Sudden death is most common in cases of aortic regurgitation where there is good compensation; when compensation fails and dropsy supervenes sudden death is uncommon. Hæmoptysis from heart failure—most commonly met with in mitral stenosis and aortic regurgitation—never causes death and may actually be beneficial. Fatty overgrowth—generally in intemperate and obese people—often leads to sudden death. On the other hand fatty degeneration is much less dangerous. Tachycardia, without any apparent reason, is a very fatal disease, it generally ends in hypertrophy, sometimes in dilatation and not unfrequently in sudden death. In compensated cases no medicinal treatment is needful, a quiet and reasonable life should be led, and a scanty dry diet with a little alcohol should be advised. If compensation fails cases of mitral regurgitation are more amenable than other valvular lesions.

**Nauheim Treatment.**—Thorne[2] gives a classification of cases of heart disease based on their suitability or not for the Nauheim treatment—a treatment which, as he had previously shown, can be quite well carried out at home. The cases which will be either cured or greatly benefited are: dilatation of the heart from influenza, typhoid fever, malaria, excessive smoking, and due to the raised arterial tension of the gouty diathesis. The cases which will be benefited are those of valvular disease with commencing cardiac failure. Unsuitable cases are: habitually heavy drinkers, cases of syphilitic disease of the heart, those with marked degeneration of the vessel walls, those with typical aortic regurgitation, and very old people.

**Arterial Hypertension.**—Huchard[3] lays considerable stress on the state of presclerosis—*i.e.*, arterial hypertension existing in a very early stage previous to any vascular or cardiac lesion. This condition in time leads to cardiac failure and dilatation. The measures which may be taken to reduce such hypertonus are dietetic, physical and drug treatment. In the first place a milk and vegetable diet is of great importance, ordinary diet is too rich in meat. In the second and third classes come diuretics, massage, and forced movements. A course at a thermal spring and such places as Bourbon-Lancy, Evian, Martigny, and Vittel are especially indicated. Gaseous baths such as are given at Nauheim are contraindicated. Amongst drugs which are useful to reduce arterial tension are iodides, trinitrin, erythrol tetranitrate, and nitrate of sodium. Thyroid extract must be used with caution. Blondel's serum, viz., the serum of fresh milk obtained by filtration after coagulation and neutralisation, is also of considerable use in reducing arterial tension. Robertson[4] says that the waters of Bourbon-Lancy are of particular service in the early stages of arterio-sclerosis with increased arterial tension, also in recent rheumatic endocarditis and in functional disease of the heart such as palpitation and paroxysmal tachycardia. On the other hand cases of cardiac failure from overstrain and from valvular disease are those which yield the most brilliant results at Nauheim. Bourbon-Lancy is a place for cardiopathies which begin in the arteries to finish in the left venticle, Nauheim for cardiopathies which commence at the heart and finish in the vessels.

[1] Clin. Jour., Feb. [2] Lancet, July 18. [3] Ibid. [4] Brit. Med. Jour., Aug. 13.

*(To be continued.)*

